# Supplementary figures and images for: Color components determination and full-length comparative transcriptomic analyses reveal the potential mechanism of carotenoid synthesis during Paphiopedilum armeniacum flowering
Source: PeerJ. 2024 Feb 22;12:e16914. doi: 10.7717/peerj.16914 (PMC10894592; doi:10.7717/peerj.16914)

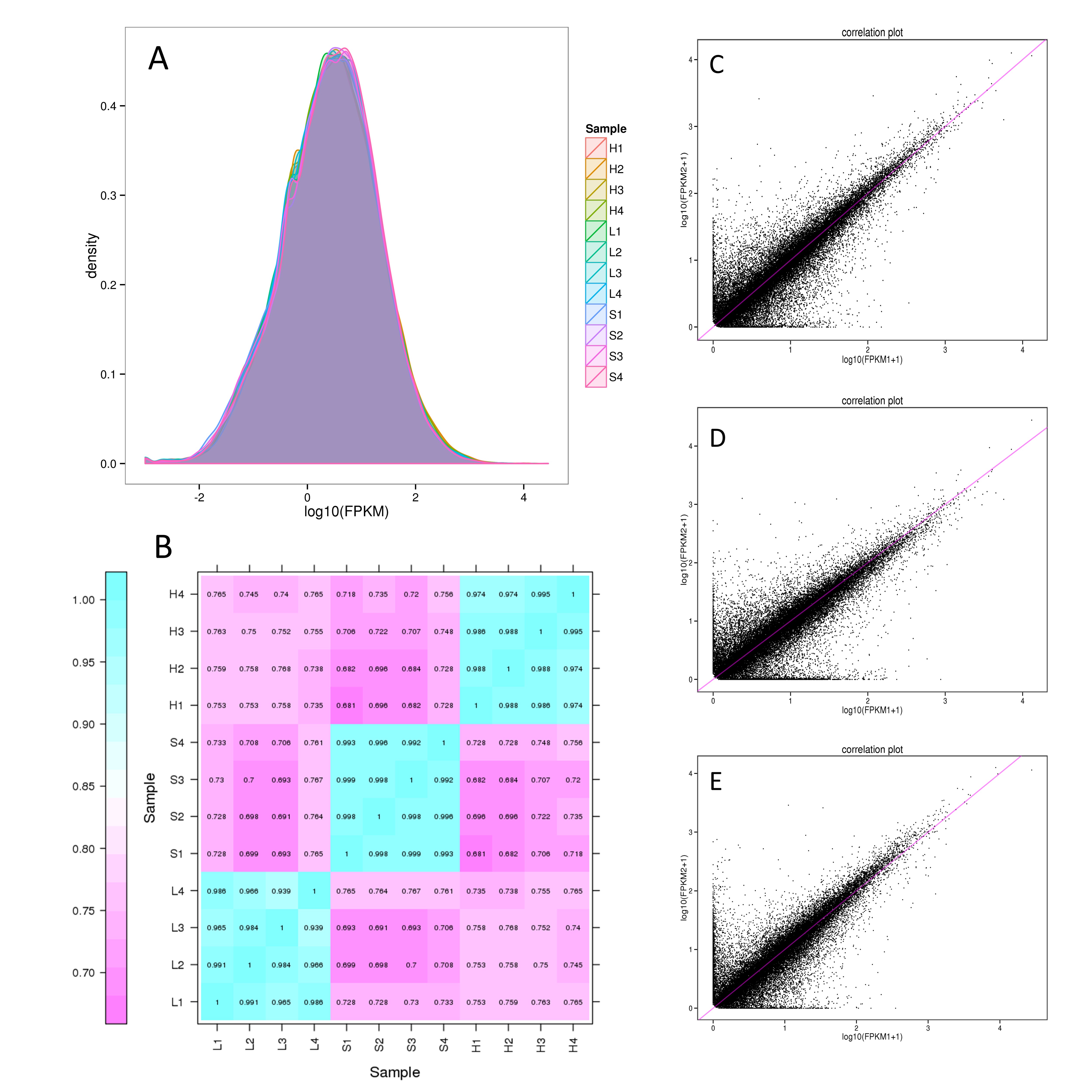

Supplement: Supplemental Information 2 — (A) Gene expression peaks for each sample; (B) Heat map depicting sample correlation; and (C–E) Scatter plots illustrating the correlation of gene expression between different groups, with (C) representing L vs H, (D) representing L vs S, and (E) representing S vs H. [file peerj-12-16914-s002.jpg]
